# Synthesis and characterization of a isothiouronium-calix[4]arene derivative: self-assembly and anticancer activity

**DOI:** 10.3762/bjoc.21.195

**Published:** 2025-11-14

**Authors:** Giuseppe Granata, Loredana Ferreri, Claudia Giovanna Leotta, Giovanni Mario Pitari, Grazia Maria Letizia Consoli

**Affiliations:** 1 CNR-Institute of Biomolecular Chemistry, Via Paolo Gaifami 18, 95126 Catania, Italy; 2 Dream Factory Lab, Vera Salus Ricerca S.r.L., Via Sigmund Freud 62/B, 96100, Siracusa, Italy; 3 J4Med Lab, Via Paolo Gaifami 9, 95126 Catania, Italy

**Keywords:** anticancer agent, calixarene, nanostructure, self-assembly

## Abstract

Calix[*n*]arenes are polyphenolic macrocycles known for their remarkable synthetic versatility, which supports their broad application in various areas, including drug discovery. Their unique conformational features, functionality, and low toxicity make calixarene derivatives valuable drug candidates against cancer. The aim of the present study was the synthesis and characterization of a calix[4]arene derivative in which known anticancer isothiouronium groups were clustered on a calix[4]arene scaffold bearing long C12 alkyl chains at the lower rim. The resulting amphiphilic calix[4]arene derivative **3** spontaneously self-assembled into nanoscale aggregates in aqueous medium, as demonstrated by dynamic light scattering analysis. The cytotoxicity of compound **3** towards cancer cells was assessed using human renal carcinoma cells (786-O cells) and compared with that in non-malignant fibroblast cells (SW1 cells). Compound **3** showed a significantly greater antiproliferative effect on cancer cells (IC_50_ 37.4 µM) than on normal fibroblasts (517 µM). The importance of the isothiouronium moieties in the observed cytoxic effect was confirmed by comparison with the calix[4]arene precursor (**1**) lacking these functional units. The selective antiproliferative profile of compound **3** highlights its potential as a lead anticancer agent. Moreover, compound **3** holds promise for further development in combination multidrug therapy due to the potential to load drug molecules in the bioactive nanoassembled structure.

## Introduction

Cancer remains one of the leading causes of morbidity and mortality worldwide. Despite significant advancements in chemotherapy, more effective and less toxic, targeted treatments are still urgently needed [1]. Among the most promising avenues in cancer therapy research, multivalent compounds have emerged as a powerful class of agents for tumor diagnosis, analysis, and therapy [2]. By exposing multiple functional groups within a single molecular framework, these compounds offer the potential for enhanced therapeutic efficacy through a multivalency effect. Multivalency is a strategy employed by nature to improve selectivity, specificity, and avidity in molecular recognition events responsible for both physiological and pathological processes. Multiple ligand–receptor interactions can significantly enhance receptor binding affinity and cellular uptake, as well as more effectively modulate signal transduction pathways, for instance when receptor clustering is necessary on the cell membrane [3]. Designing multivalent bioactive compounds thus represents a promising approach to overcoming several limitations associated with traditional monovalent therapeutics, including drug resistance, off-target effects, high dosage requirements, and insufficient specificity. This strategy may pave the way for next-generation anticancer therapies more effective and precise than current treatments. Numerous multivalent constructs have been reported in literature, both as compounds with intrinsic anticancer activity [4] and nanocarriers for anticancer drug delivery [5–6].

In the development of multivalent compounds, macrocyclic scaffolds such as cyclodextrins [7], cyclic peptides [8], cucurbiturils [9], resorcinarenes [10], pillarenes [11], prismarenes [12], and calix[*n*]arenes have proven to be particularly valuable.

Calix[*n*]arenes, a family of polyphenolic macrocyclic oligomers, are widely utilized in applications ranging from materials science to life sciences [13–17]. Their synthetic versatility, ease of functionalization, conformational rigidity or flexibility, steric bulk, and low cost make them highly attractive in the development of novel, non-conventional anticancer agents with a great potential for overcoming the toxicity of cancer chemotherapy and achieving targeted treatments [18–20].

In the era of nanotechnology, a promising strategy for developing more effective treatments against cancer [21–22], calixarene derivatives are also emerging as valid building blocks for the development of nanoscale multivalent constructs [23]. Due to the nanosize, the calixarene-based nanoconstructs could preferentially accumulate in cancer tissues by exploiting the enhanced tumor permeability and retention (EPR) effect [24] or selectively penetrate cancer cells through specific ligand–receptor interactions on the surface of target cells [25–26]. A variety of calixarene-based nanoconstructs have been reported, including nanosystems with light-triggered anticancer activity [27], potential anticancer vaccine candidates [28], and nanocarriers for anticancer drug delivery [29–33].

Numerous bioactive groups have been introduced into the calixarene skeleton to develop anticancer derivatives, including proline [34], carbonyl amide [35], glycoureido [36], ureido [37], picolylamine [38], 5-bromopentyltrimethylammonium bromide and 3-bromopropyltriphenylphosphonium bromide [39], among others.

Isothiouronium salts represent another class of interest in the search for new chemotherapeutic agents. They are known inhibitors of protein kinase C [40] and agonists of the GABA-type [41] and histamine-H3 receptors [42]. *S*-Allylic isothiouronium salts substituted with aliphatic groups have shown to combine high antitumor activity against leukemia cells and low toxicity toward non-cancerous cells [43].

Therefore, the aim of this study was to evaluate whether introducing isothiouronium moieties at the upper rim of a calix[4]arene scaffold, bearing long C12 alkyl chains at the lower rim, could yield a novel derivative (compound **3**) with anticancer activity. The synthesis and structural characterization of compound **3** were carried out, and its self-assembly behavior in aqueous medium was investigated using dynamic light scattering (DLS) and electrophoretic light scattering (ELS). The anticancer potential of compound **3** was assessed in human renal carcinoma cells (786O) and normal human fibroblasts (SW1), and the tumor cells selectivity was evaluated. To elucidate the role of the isothiouronium groups, antiproliferative effects were also estimated on calix[4]arene precursor **1** lacking these functional groups.

## Results and Discussion

### Synthesis and characterization of compound **3**

**Scheme 1:**
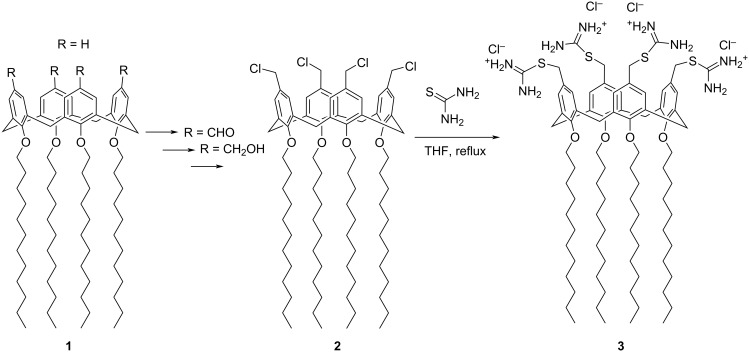
Procedure for the synthesis of compound **3**.

For the synthesis of isothiouronium-calix[4]arene derivative **3** (Scheme 1), the chloromethylcalix[4]arene derivative **2** was prepared by adopting a procedure reported in the literature [44].

In brief, starting from the commercial *p*-H-calix[4]arene **1**, four C-12 alkyl chains were tethered to the calixarene hydroxy groups (lower rim) by reaction with dodecyl iodide in the presence of NaH. At the upper rim of this derivative, four formyl groups were introduced by reaction with hexamethylenetetramine in trifluoroacetic acid. The formyl groups were reduced to alcohol groups by sodium borohydride and then converted to chloromethyl groups by treatment with thionyl chloride, to give compound **2**.

**Figure 1:**
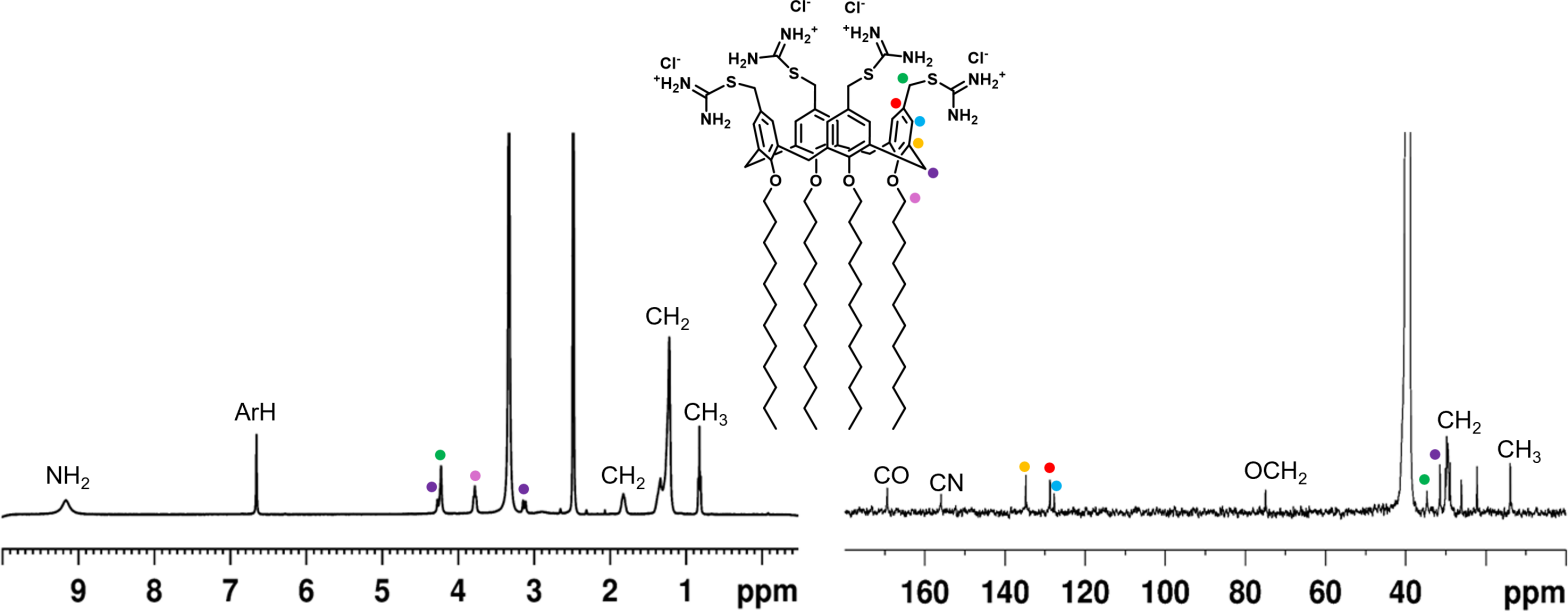
^1^H NMR (left) and ^13^C NMR (right) spectra of compound **3** in DMSO-*d*_6_ (400.13 MHz, 297 K).

The reaction of compound **2** with thiourea in THF gave compound **3** as a white solid in 96% yield. Compound **3** was characterized by ^1^H and ^13^C NMR in DMSO-*d*_6_ as a solvent (Figure 1). The proton signals of the NH₂ and CH₂S groups of the isothiouronium substituents were clearly observed at 9.04 ppm and 4.23 ppm, respectively. Additionally, the carbon signal of the CH₂S group appeared at 169.3 ppm. The NMR signals, consistent with a fully symmetric structure, evidenced the exhaustive functionalization of the calix[4]arene skeleton blocked in cone conformation, as evidenced by the AX system (3.13 and 4.26 ppm) for the bridged CH_2_ protons.

### Spontaneous self-assembly

**Figure 2:**
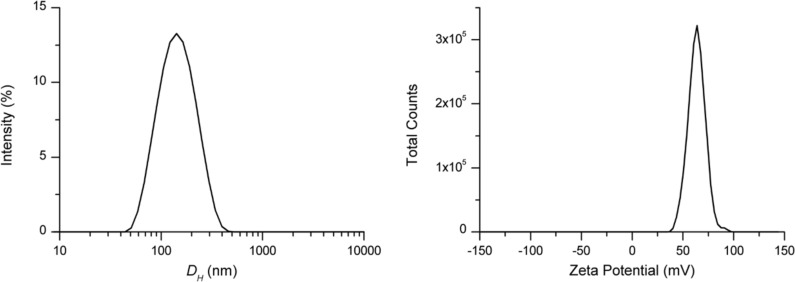
Intensity-weighted mean hydrodynamic diameter (left), and zeta potential distribution (right) of compound **3** (1 mg/mL water), after vortex and sonication.

Due to the amphiphilic nature, compound **3** could spontaneously self-assemble in aqueous medium. This was confirmed by dynamic light scattering measurements, which showed the presence of nanoaggregates with mean hydrodynamic diameters of 125 ± 1 nm in the aqueous colloidal solution of compound **3** (Figure 2).

A polydispersity index of 0.18 ± 0.01 was indicative of a good dimensional homogeneity of the nanoaggregates in the suspension.

**Figure 3:**
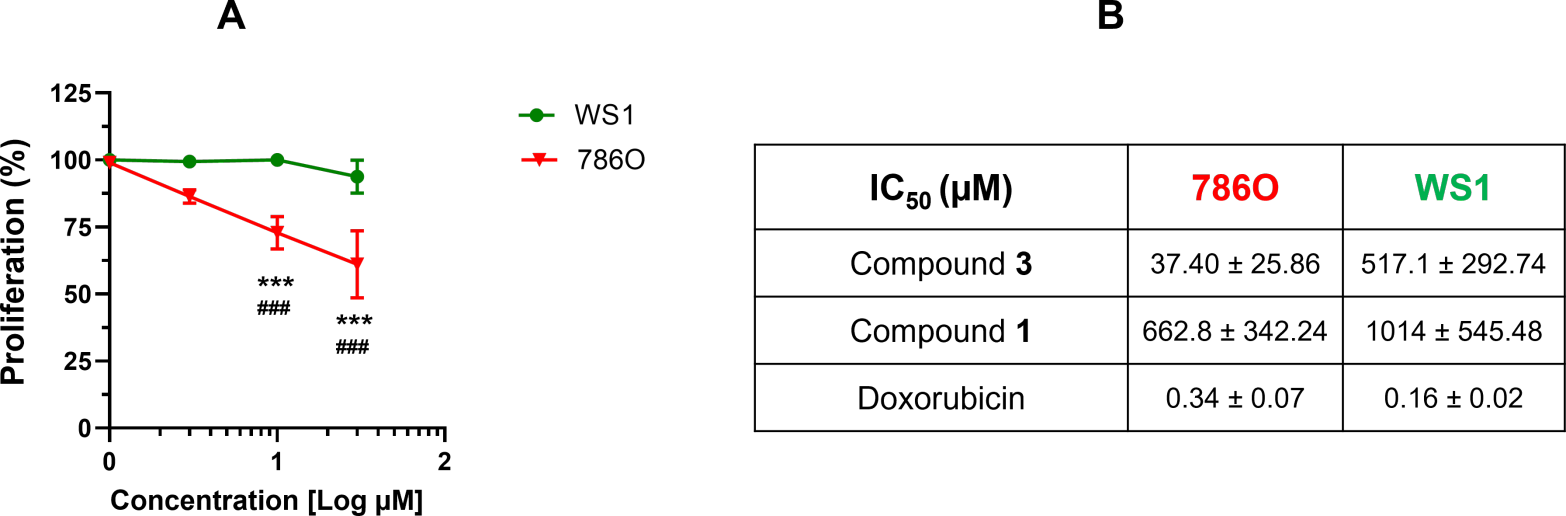
Antiproliferative effects by compound **3** and its chemical precursor **1**. A) Results on proliferative kinetics in renal cancer 786O and skin fibroblast WS1 cells. Data represent the percentage of proliferation with respect to the correspondent vehicle DMSO controls. ***, *p* < 0.001 vs the respective vehicle control by 2-way ANOVA; ###, *p* < 0.001 vs correspondent WS1 conditions by 2-way ANOVA. B) Calculated IC_50_ for compounds **3** and **1** in renal cancer 786O and skin fibroblast WS1 cells. Doxorubicin, the positive control. Data are expressed as means ± SD.

The zeta potential is a measure of the electrical potential between the surface of nanoaggregates and the surrounding fluid. Electrophoretic light scattering (ELS) provided for the nanoaggregates of compound **3** a zeta potential (ζ) value of +64 ± 1 mV (Figure 3), indicative of stable nanoassemblies. Indeed, it is generally accepted that values higher than ±40 mV are associated with strong stability by repulsion, preventing further aggregation phenomena.

### Cytotoxicity and cancer cell selectivity

Thiouronium-containing molecules can inhibit cancer cells, inducing apoptosis or necrosis, by different mechanisms including elevation of reactive oxygen species (ROS) or interference with redox homeostasis, and inhibition of kinase or topoisomerase activities essential for cancer cell proliferation. The selective activity of thiouronium salts against cancer cells compared to non-malignant cells can be related to a specific sensitivity of cancer cells [43]. In fact, cancer cells operate under higher oxidative stress than normal cells and often overexpress kinases or topoisomerases, which make them more vulnerable than normal cells.

The anticancer effect of compound **3** was assessed on human renal carcinoma 786O cells, through evaluation of proliferative kinetics with the acridine orange staining assay [45]. The 786O cell line is a widely used in vitro model for studying clear cell renal cell carcinoma (ccRCC) [46], which accounts for over 90% of all renal malignancies and is characterized by poor responsiveness to conventional chemotherapy.

To the best of our knowledge, calixarene derivatives have not been previously tested as anticancer agents against human renal carcinoma cells.

Compound **3** suppressed the proliferation of the renal cancer cells (Figure 3) with a calculated 50% inhibitory concentration (IC_50_) of 37.40 µM. In contrast, compound **3** was poorly effective against normal WS1 fibroblasts (Figure 3), exhibiting an IC_50_ value of 517 µM. These values yielded a selectivity index (SI) of approximately 14, indicating that tumor cells are significantly more sensitive to the cytotoxic effects of compound **3** than healthy, non-malignant cells. In general, SI values above 12 are associated to remarkable selectivity, 6–12 to moderate selectivity and 1–5 to weak selectivity [43,47]. In contrast, the potent antineoplastic agent doxorubicin, used as the positive control for anticancer effects, did not discriminate between normal and cancer cells, as demonstrated by the calculated SI of 0.47. The high SI value by compound **3** evidenced the isothiouronium moieties retain the selective cytotoxicity towards cancer cells also when tethered to the calixarene scaffold.

Since the long alkyl chains in compound **3** could be responsible for molecular disruptive events at the cell membrane through intercalation in the lipidic bilayer, the effects of *p*-H-calix[4]arene-*O*-dodecyl derivative **1**, a precursor lacking isothiouronium moieties, on cell proliferation were also investigated. The absence of the isothiouronium groups substantially blunted the antiproliferative activity in both cancer and normal cells, as demonstrated by the higher IC_50_ values exhibited by compound **1** (Figure 3). These data clearly demonstrated that isothiouronium moieties are crucial for the anticancer activity of compound **3**.

## Conclusion

The introduction of four isothiouronium functional groups at the upper rim of a calix[4]arene macrocycle, bearing four dodecyl aliphatic chains at the lower rim, yields an amphiphilic derivative that spontaneously self-assembles into nanoscale structures with a mean hydrodynamic diameter of approximately 100 nm and a positively charged surface (zeta potential + 64 mV). These values are suitable for potential applications in the field of nanomedicine. Remarkably, the isothiouronium-calix[4]arene derivative, unlike its precursor lacking isothiouronium functionalities, inhibited the proliferation of human renal carcinoma cells with notable selectivity over normal human fibroblasts. To the best of our knowledge, this is the first study evaluating the antiproliferative activity of both a thiouronium salt and a calix[4]arene derivative in renal carcinoma cells. The isothiouronium-calix[4]arene nanoassemblies may be promising candidates for further investigations as a drug delivery system. The entrapment of different drugs combined with the intrinsic anticancer activity of the nanoassemblies could provide new agents for a combination multidrug anticancer treatment.

## Experimental

**Materials and methods:** All chemicals were purchased from Sigma-Aldrich (Milan, Italy) and used without further purification. Human renal adenocarcinoma 786-O cells were obtained from the American Type Culture Collection (ATCC; Manassas, VA, USA). Cells (passages 2-10) were maintained at 37 °C (5% CO_2_) in RPMI 1640 medium, containing 10% FBS, 2 mM ʟ-glutamine, 100 units/mL penicillin and 100 µg/mL streptomycin. All cell media and reagents were from Euroclone S.p.A. (Pero, Milan, Italy).

**Procedure for the synthesis of compound 3:** Compound **3** was synthesized from the chloro-methyl-calix[4]arene precursor **2** [44] adopting a procedure described for the synthesis of an analogous tetrakis(thiuroniumethyl)tetramethoxycalix[4]arene tetrachloride [48]. Compound **2** (100 mg, 0.077 mmol) and thiourea (1.5 equiv per chloromethyl unit) were dissolved in THF (4 mL) and the solution was refluxed for 15 h to give a white precipitate. After cooling, the solid precipitate was recovered by filtration and washed several times with ethyl ether, to give pure compound **3** (118 mg, 96% yield).

**Characterization of compound 3:** NMR spectra were acquired on a Bruker Avance 400TM spectrometer. Chemical shifts (δ, ppm) are reported referring to the residual peak of the solvent (DMSO-*d*_6_). ^1^H NMR (400.13 MHz, DMSO-*d*_6_, 297 K) δ 0.83 (t, *J* = 6.6 Hz, 12H, 4 × dodecyl CH_3_), 1.22 (br s, 72H, 36 × dodecyl CH_2_), 1.34 (br s, 8H, 4 × dodecyl CH_2_), 1.82 (t, 8H, 4 × dodecyl CH_2_), 3.13 (d, *J* = 14.3 Hz, 4H, 2 × ArCH_2_Ar), 3.78 (t, 8H, 4 × dodecyl OCH_2_), 4.23 (s, 8H, 4 × ArCH_2_S), 4.26 (d, *J* = 14.3 Hz, 4H, 2 × 4 ArCH_2_Ar), 6.66 (s, 8H, 8 × ArH); ^13^C NMR (100.6 MHz, DMSO-*d*_6_, 297 K) δ 13.9 (q, dodecyl CH_3_), 22.2, 26.2, 29.0, 29.5, 29.8, 30.1 (t, dodecyl _CH2_), 31.5 (t, ArCH_2_Ar), 34.7 (t, CH_2_S), 75.0 (t, OCH_2_), 127.8 (d, ArH), 128.8 (s, ArC-_CH2S_), 135.0 (s, ArC-CH_2_S), 156.0 (s, ArCO), 169.4 (s, C-NH_2_).

**Size and zeta potential measurements:** DLS and ELS measurements were performed on a sample of compound **3** (1 mg) dissolved in 1 mL pure water. The colloidal solution was centrifuged at 10,000 rpm for 10 min and the supernatant was collected and analyzed by a Zetasizer NanoZS90 analyzer (Malvern Instrument, Malvern, UK) equipped with a 633 nm laser, at a scattering angle of 90° and 25 °C temperature. The size of the particles was calculated from the diffusion coefficient using the Stokes–Einstein equation:


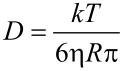


where *D* is the diffusion coefficient, *k* is the Boltzmann constant, *T* is the absolute temperature, η is the solvent viscosity, and *R* is the solute radius.

The zeta potential (*Z*) was calculated by using Henry’s equation


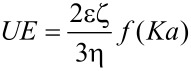


where *UE* is the electrophoretic mobility, ζ is the dielectric constant, *f*(*Ka*) is the Henry’s function, and η is the viscosity.

**Biological assays:** Cell viability was measured by the acridine orange staining assay. Briefly, cancer cells were seeded in 96-well plates and grown at optimum culture conditions for 72 h (at a confluence of ≈60–80%). Then, cells were treated with the indicated concentrations of compounds for additional 48 h. Control cells received an equal volume of vehicle (DMSO). At the end of incubations, cells were fixed (in 4% paraformaldehyde) and stained with acridine orange solution (50 µg/mL), as previously described [45]. Acridine orange staining was then quantified as the resulting fluorescence intensity (excitation 485/20 nm, emission 528/20 nm) with a microplate reader (Synergy HT, BioTek).

**Statistical analyses:** Results are shown as mean ± SEM of three independent experiments, performed at least in triplicate. Statistical comparisons were performed by 2-way ANOVA. *P* values were considered significant at *p* ≤ 0.05. All analyses were done with GraphPad Prism 10.4.1 (GraphPad Software, Inc., San Diego, CA).

**Selectivity index determination:** The selectivity index of compound **3** was calculated as the ratio of the IC_50_ in the cancer cell line to the IC_50_ in the non-malignant cell line, according to the following formula


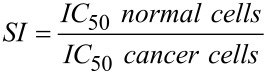


## Data Availability

All data that supports the findings of this study is available in the published article and/or the supporting information of this article.
